# Removal of toxic metals from aqueous solution by biochars derived from long-root *Eichhornia crassipes*

**DOI:** 10.1098/rsos.180966

**Published:** 2018-10-24

**Authors:** Qiang Li, Lizhou Tang, Jiang Hu, Ming Jiang, Xiaodong Shi, Tianxi Zhang, Yuan Li, Xuejun Pan

**Affiliations:** 1 Faculty of Biological Resources and Food Engineering, Qujing Normal University, Qujing, People's Republic of China; 2 Key Laboratory of Yunnan Province Universities of the Diversity and Ecological Adaptive Evolution for Animals and Plants on Yun-Gui Plateau, Qujing Normal University, Qujing, People's Republic of China; 3 College of Resources and Environment, Yunnan Agricultural University, Kunming, People's Republic of China; 4 Faculty of Environmental Science and Engineering, Kunming University of Science and Technology, Kunming, People's Republic of China

**Keywords:** toxic metal, biochar, long-root *Eichhornia crassipes*, adsorption

## Abstract

Biochars were produced from long-root *Eichhornia crassipes* at four temperatures: 200, 300, 400 and 500°C, referred to as LEC200, LEC300, LEC400 and LEC500, respectively. The sorption ability of lead, zinc, copper and cadmium from aqueous solutions by four kinds of biochars was investigated. All the biochars had lower values of CEC and higher values of pH. LEC500 was the best one to bind toxic metals which can be reflected in the results of SEM, BET and elemental analyser. It was also found that alkyl, carboxyl, phosphate and cyano groups in the biochars can play a role in binding metals. In addition, the sorption processes of four metals by the biochars in different metal concentration were all excellently represented by the pseudo-second-order model with all correlation coefficients *R*^2^ > 0.95. And the sorption processes of four metals in different temperatures could be described satisfactorily by the Langmuir isotherms. According to calculated results by the Langmuir equation, the maximum removal capacities of Pb(II), Zn(II), Cu(II) and Cd(II) at 298 K were 39.09 mg g^−1^, 45.40 mg g^−1^, 48.20 mg g^−1^ and 44.04 mg g^−1^, respectively. The positive value of the Δ*H*^0^ confirmed the adsorption process was endothermic and the negative value of Δ*G*^0^ confirmed the adsorption process was spontaneous. The sorption capacities were compared with several other lignocellulosic materials which implied the potential of long-root *Eichhornia crassipes* waste as an economic and excellent biosorbent for eliminating metal ions from contaminated waters.

## 1. Introduction

Toxic metal pollution is a grave environmental problem now because toxic metals, such as lead, zinc, copper and cadmium, are among the most common pollutants found in industrial effluents. Many industrial activities such as metal plating, the fertilizer industry, mining operations and textiles introduce toxic metals to the environment via their waste effluents [1]. These toxic metals are not biodegradable and tend to accumulate in living organisms, causing various diseases and disorders [2]. Therefore, they must be removed from the aqueous solution before discharging. Currently, various methods or technologies, such as chemical precipitation, reverse osmosis, electrodialysis, coagulation, ion exchange and adsorption have been used to remove heavy metal ions from aqueous solutions [3–9].

Among these treatment procedures, sorption as one of the most widely used methods for toxic metal removal has been given more and more attention owing to its high removal efficiency and clean process. Research in recent years has also indicated that some natural biomaterials can accumulate high concentrations of heavy metals in their body such as walnut shells [10], dry tree leaves [11], rice hulls [12], dairy manure [13], saw dust [14] and corn cobs [15]. Biochar is a carbonaceous solid residue of thermal treatment of carbon-rich biomass under O_2_-limited and low temperatures, a process known as low-temperature pyrolysis [16]. The use of biochar as a low-cost sorbent to remove metallic contaminants from aqueous solutions is an emerging and promising wastewater treatment technology, which has already been demonstrated in previous studies [17–27]. Aquatic macrophytes have large surface areas, and their living bodies have good performance on heavy metal absorption [28–30]. Many studies indicated that *Eichhornia crassipes* could remove heavy metals efficiently [31–33]. *Eichhornia crassipes* can survive in extreme conditions and tolerate very high concentrations of heavy metal [34–36]. Furthermore, a number of researchers have studied the adsorption of heavy metals from aqueous solution by dehydrated powders of *Eichhornia crassipes* [28,37]. However, according to the authors' survey, there is no extensive study on the adsorption of heavy metals from solutions using biochars from *Eichhornia crassipes* in the literature, let alone long-root *Eichhornia crassipes*.

The objective of this study was to examine the efficiencies of four kinds of biochars derived from long-root *Eichhornia crassipes* for Pb(II), Zn(II), Cu(II) and Cd(II) removal from aqueous solutions. The surface properties of the biochars such as CEC and pH values were investigated. Furthermore, biochars of LEC200, LEC300, LEC400 and LEC500 were compared for their potentially different physical and chemical characteristics (BET surface area and total pore volume, pore diameter, Fourier transform infra-red (FT-IR) spectroscopy, elemental analyser and scanning electron microscope). Lastly, the effect of adsorbent dosage on adsorption, contact time and initial metal concentration on adsorption kinetics, adsorption isotherms and thermodynamic were investigated.

## 2. Material and methods

### 2.1. Pyrolysis experiments and biochar samples

The biochars used in this study were from the root powders of long-root *Eichhornia crassipes.* Firstly, the roots of live long-root *Eichhornia crassipes* were cut down, washed with tap water followed by distilled water to eliminate mud, dried at 105°C for 0.5 h and 65°C to constant weight, grounded to powders to make the root powders. The root powders were then placed in a Muffle furnace and heated under an O_2_-limited condition at 200, 300, 400 and 500°C. After 4 h heating, the furnace was turned off and the samples were allowed to cool to room temperature. The solid residue left in the reactor was designated as biochar and those produced from the root powders at 200, 300, 400 and 500°C were referred to as LEC200, LEC300, LEC400 and LEC500, respectively. The biochars were grounded and passed through a 1 mm sieve for characterization and sorption experiment.

### 2.2. Reagents and apparatus

All the chemical reagents used in this study were analytically pure grade. The Pb(II), Zn(II), Cu(II) and Cd(II) stock solutions were prepared by dissolving Pb(NO_3_)_2_, Zn(NO_3_)_2_, Cu(NO_3_)_2_ and Cd(NO_3_)_2_ in deionized water, respectively. All glassware was soaked in 15% HNO_3_ then rinsed with deionized water before use. A flame atomic absorption spectrometer (FAAS, Varian Instruments AA240FS) was used for sample analysis in this study.

### 2.3. Surface properties and morphology

The surface morphology of the root powders before and after adsorption was determined by SEM and energy spectrum analysis (FEI QUANTA200). The pore diameter and surface area were determined by the BET method. An FT-IR (Varian 640-R) was used to analyse the change of functional groups before and after adsorption. An LECO elemental analyser was used to determine carbon, hydrogen, oxygen, nitrogen and sulfur content in the biochars.

### 2.4. Batch adsorption experiments

The recovery ratios and CEC values of LEC200, LEC300, LEC400 and LEC500 were determined before the experiments. Then the four kinds of biochars with the quantities of 0.1, 0.5, 1, 2 and 3 g l^−1^ were added to the flasks containing Pb(II), Zn(II), Cu(II) and Cd(II) aqueous solutions at concentration of 100 mg l^−1^ to investigate the optimum quantity. Different metal concentrations of 10, 20 and 30 mg l^−1^ were prepared and samples taken at *t* = 1, 3, 5, 7, 9, 12, 15, 17, 20, 25, 30, 40, 60, 90 and 120 min to study the adsorption kinetics. LEC500 of 1 g l^−1^ was added to the metal solutions to research the adsorption isotherms at different temperatures of 298, 308 and 318 K. In batch adsorption experiments, four kinds of biochars were added to the flasks which were placed in an oscillation at 140 rpm. After equilibrium, solid and liquid phases were separated by centrifugation at 4000 rpm for 15 min and the solution was filtered through a 0.45 μm hybrid fibre membrane, and the concentrations of heavy metal were determined by FAAS.

### 2.5. Data analysis

For the adsorption treatments, results from three replicate samples were averaged, and control samples were set for analysis. Langmuir and Freundlich adsorption models were used to describe the metal absorption process. The Langmuir model can be expressed in the following form:

2.1 $$q_{e}=\frac{C_{e}bq_{\max}}{1+C_{e}b},$$

where *C_e_* is the equilibrium concentration (mg l^−1^), *q_e_* and *q*_max_ are the equilibrium and maximal adsorption capacity (mg g^−1^), respectively, and *b* is the equilibrium constant [38].

The Freundlich model can be expressed in the following form:

2.2 $$q_{e}=K_{F}C_{e}^{1/n},$$

where *K*_F_ is the Freundlich coefficient (mg g^−1^), indicating adsorption capacity; *n* is the Freundlich index, indicating degrees of adsorption [39].

First-order and second-order equations are used to describe the kinetics of heavy metal adsorption [40].

The linear form of first-order rate equation is as follows:

2.3 $$q_{t}=q_{e}(1-e^{-K_{1}t}),$$

where *K*_1_ is the first-order rate constant (min^−1^), and *q_e_* and *q_t_* are the amount of metal adsorbed per unit weight of adsorbent (mg g^−1^) at equilibrium and at time *t*, respectively. The linear form of the second-order equation is

2.4 $$q_{t}=\frac{K_{2}q_{e}^{2}t}{1+K_{2}q_{e}t},$$

where *K*_2_ is a pseudo-second-order equilibrium constant (g mg min^−1^).

Thermodynamic parameters such as Gibb's free energy (Δ*G*^0^), enthalpy change (Δ*H*^0^) and change in entropy (ΔS^0^) for the adsorption of metals on biomass have been determined by using the following equations:

2.5 $$\Delta G^{0}=\Delta H^{0}-T\Delta S^{0},$$

2.6 $$\Delta G^{0}=-RT\ln\left(\frac{q_{e}}{C_{e}}\right)$$

2.7 $$\;\mathrm{and}\;\log\left(\frac{q_{e}}{C_{e}}\right)=\frac{\Delta S^{0}}{R}+\frac{-\Delta H^{0}}{RT},$$

where *C_e_* is the equilibrium concentration (mg l^−1^), *q_e_* is the equilibrium and maximal adsorption capacity (mg g^−1^) and *T* is temperature in K and *R* is the gas constant (8.314 J mol^−1^K).

## 3. Results and discussion

### 3.1. Characterization of the biochars

#### 3.1.1. Fourier transform infra-red spectroscopy analysis

**Figure 1.**
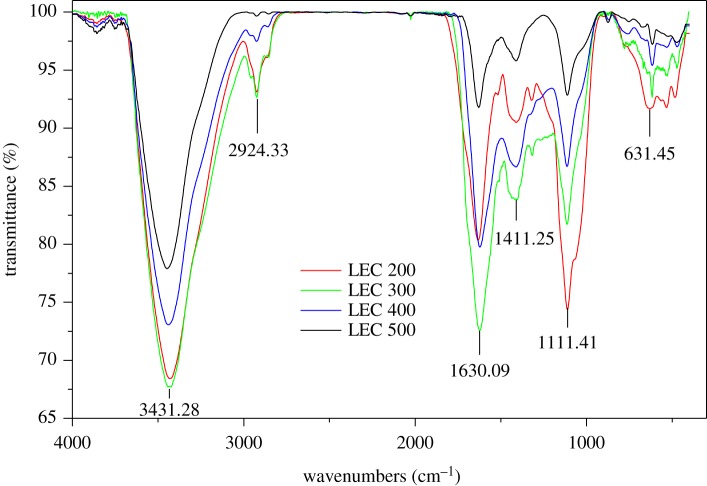
FT-IR spectra of LEC200, LEC300, LEC400 and LEC500.

FT-IR spectra of LEC200, LEC300, LEC400 and LEC500 were shown in figure 1. As shown in figure 1, the significant peaks of the four kinds of biochars on approximately 3431, 2930, 1630, 1410, 1058 and 630 cm^−1^ showed the functional groups mainly including hydroxyl, alkyl, secondary amine group, carboxyl, phosphate and cyano, and obviously, these groups decreased with the increase in temperature. However, FT-IR analysis of biochars laden metal and the fresh ones showed that a number of adsorption peaks had shifted after toxic metal sorption. For LEC200 and LEC300, alkyl, carboxyl, phosphate and cyano groups were primary sorption sites for lead, zinc, copper and cadmium binding. For LEC400 and LEC500, there was considerable change at the peaks of phosphate and cyano groups, indicating much possibility of adsorption at these sites (electronic supplementary material, figure S1).

The amounts of functional group in biochars usually decreased with the increase in temperature and some aromatic hydrocarbon appeared at a high temperature. Some literature also emphasized that the alkyl, carboxyl, phosphate and cyano groups in biochars can play a role in adsorbing metals [41].

#### 3.1.2. Biochar characterization

As expected, four kinds of biochars were rich in carbon and carbon values increased with increasing temperature according to elemental analyses (electronic supplementary material, table S1). The increase in carbon probably resulted from the increasing biomass combustion with increasing temperature. Nitrogen content of the biochars was quite similar and content of oxygen decreased with increasing temperature due to the biochars under the O_2_-limited condition. Sulfur and nitrogen groups behave as Lewis bases and contribute to carbon basicity, while oxygen and hydrogen attribute acidic properties to the carbon surfaces [42]. Thus, it was expected that LEC200 should be the most acidic one and LEC500 the most alkaline.

The specific surface areas were 1.498, 1.558, 6.212 and 8.008 m^2^ g^−1^ for LEC200, LEC300, LEC400 and LEC500, respectively (electronic supplementary material, table S2). The total pore volumes were 0.009, 0.005, 0.033 and 0.056 cm^3^ g^−1^ for LEC200, LEC300, LEC400 and LEC500, respectively. BET results showed that the specific surface area and total pore volume increased with the calcinations temperature for biochars. But the average pore diameters of the biochars were similar and around 20 nm. These indicated that LEC500 may be the best one for metal adsorption.

The productivities of LEC200, LEC300, LEC400 and LEC500 were 73.69%, 49.61%, 43.63% and 36.99%, respectively. At first, pH values of the four kinds of biochars were 7.68, 9.40, 10.26 and 10.37, respectively. After adsorption of metals, pH values decreased a lot, indicating that chemical precipitation made a contribution to adsorbing metals (electronic supplementary material, table S3). Then, MgSO_4_ was used to exchange the Ba^2+^ after the saturation adsorption of Ba^2+^ onto the biochars to determine the CEC values. It turned out that the CEC values of LEC200, LEC300, LEC400 and LEC500 were 1.04, 1.17, 1.29 and 1.28 cmol kg^−1^, respectively. These low CEC values showed that ion exchange may be not the key mechanism on adsorbing metals.

**Figure 2.**
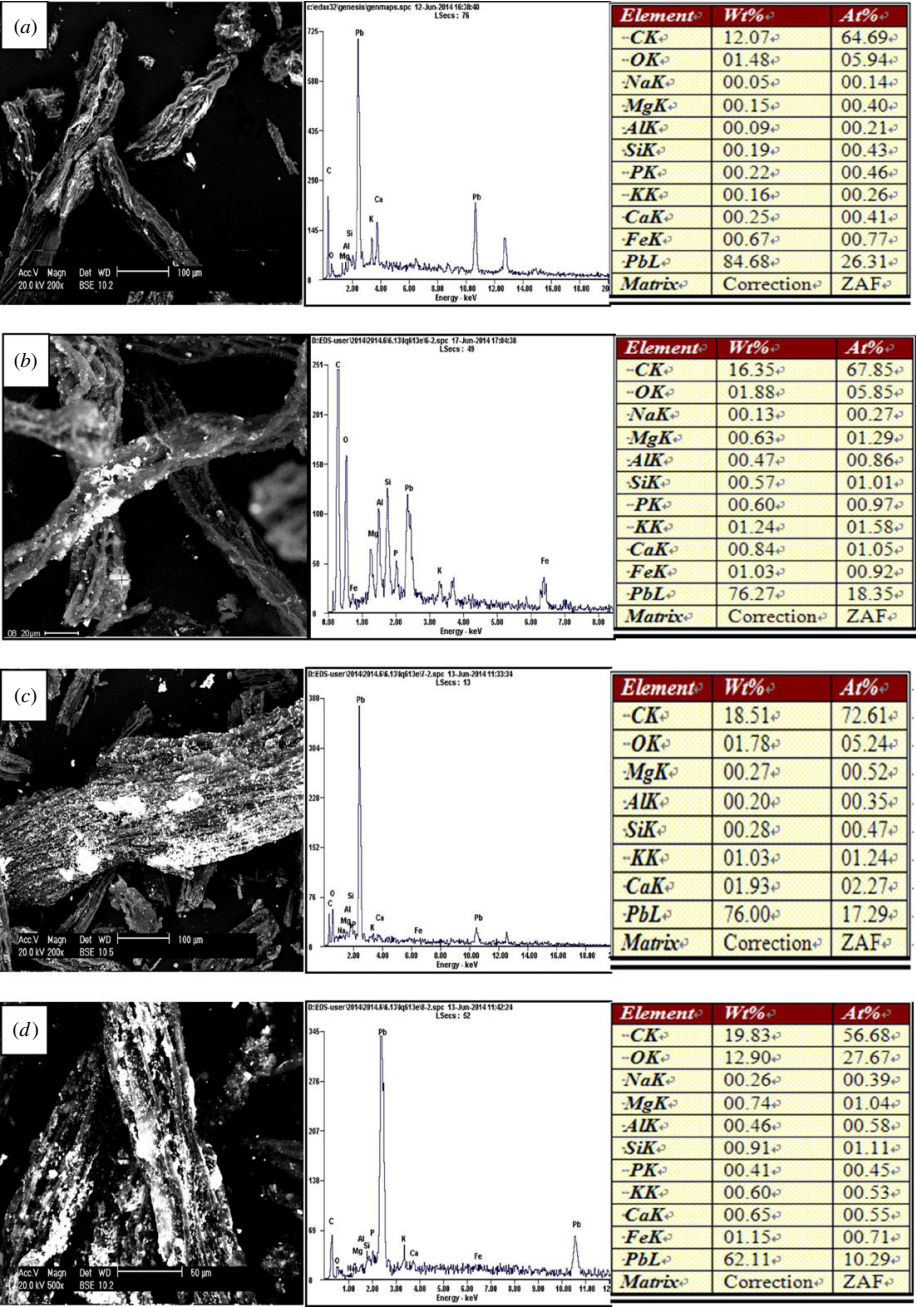
Scanning electron micrographs and energy spectrum analysis of the biochars after adsorbing Pb(II): (*a*) LEC200, (*b*) LEC300, (*c*) LEC400 and (*d*) LEC500.

**Figure 3.**
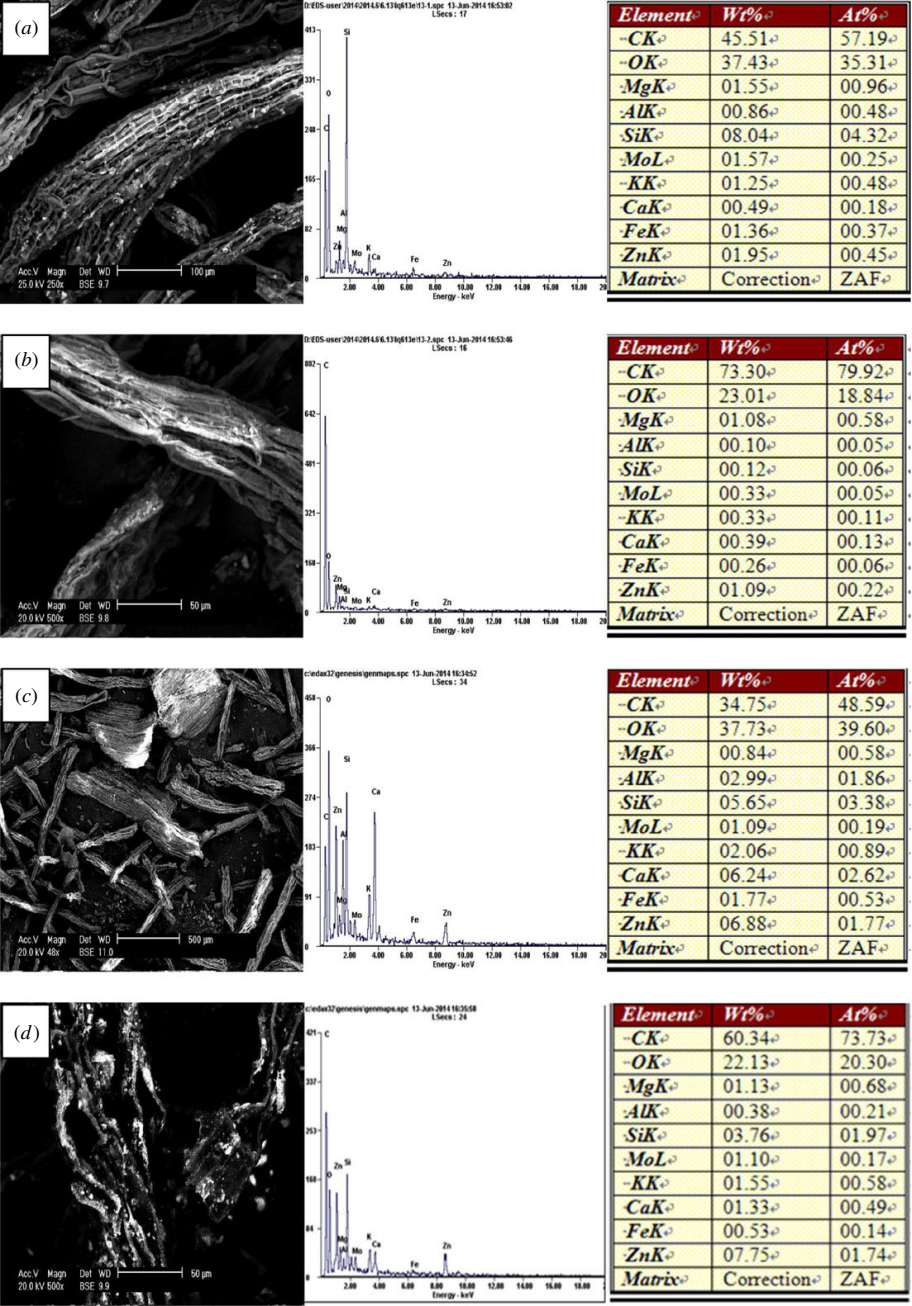
Scanning electron micrographs and energy spectrum analysis of the biochars after adsorbing Zn(II): (*a*) LEC200, (*b*) LEC300, (*c*) LEC400 and (*d*) LEC500.

**Figure 4.**
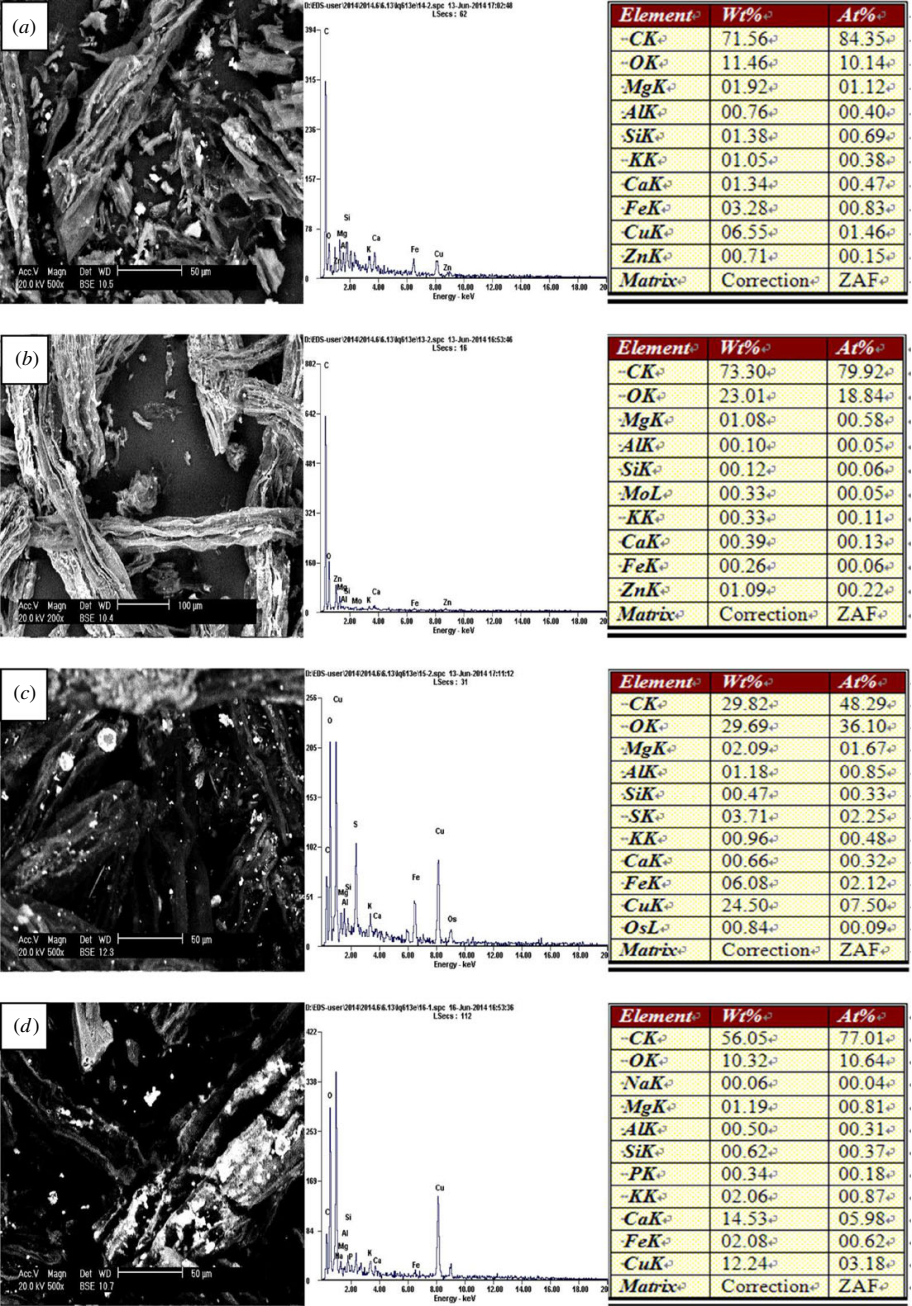
Scanning electron micrographs and energy spectrum analysis of the biochars after adsorbing Cu(II): (*a*) LEC200, (*b*) LEC300, (*c*) LEC400 and (*d*) LEC500.

**Figure 5.**
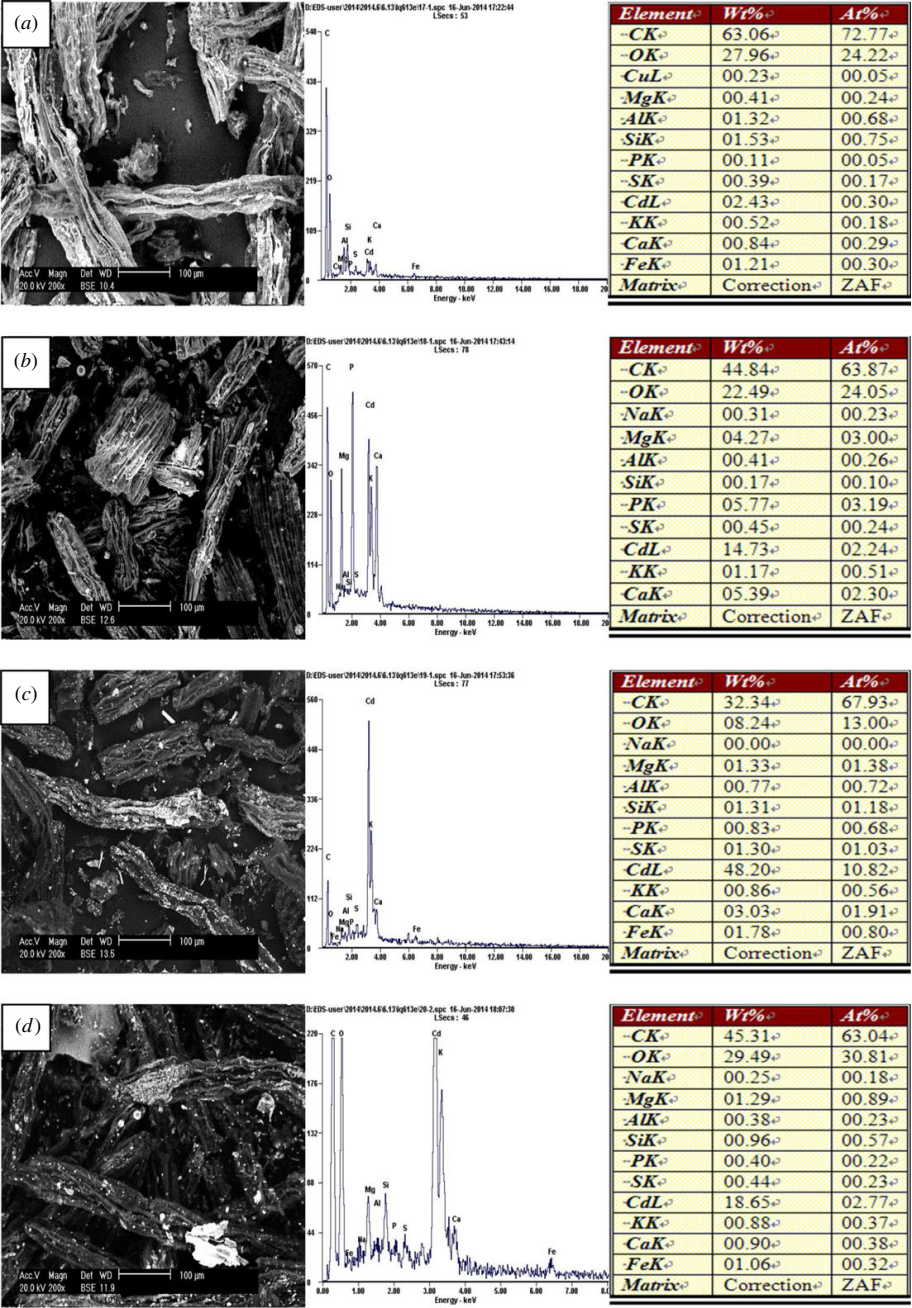
Scanning electron micrographs and energy spectrum analysis of the biochars after adsorbing Cd(II): (*a*) LEC200, (*b*) LEC300, (*c*) LEC400 and (*d*) LEC500.

Scanning electron micrographs showed that the particle did not present a clear crystal form and its surface was an irregular rectangle for LEC200, LEC300 and LEC400. The surface began to collapse thoroughly for LEC500 which was caused by the high temperature (electronic supplementary material, figure S2). The ramping procedures led to an increase in microporous and surface area which were probably conducive to adsorption. Pb, Zn, Cu and Cd were adsorbed on the surface of the biochars after experiments which can be clearly observed from the images of SEM in figures 2–5 and more metals were adsorbed on the surface with the increase in temperature. Energy spectrum analysis showed that four kinds of biochars were rich in C and O and a small number of K, Ca, Na, Mg, Al, Fe, Si, P which were common elements in aquatic macrophytes.

### 3.2. Adsorption proprieties of the biochars

#### 3.2.1. Effect of adsorbent dosage on adsorption

The effect of adsorbent dosage on adsorption was shown in the supplementary material (electronic supplementary material, figure S3). The removal percentage of Pb(II), Zn(II), Cu(II) and Cd(II) increased with the increase of adsorbent dosage, but the adsorbing capacity decreased with the increase of adsorbent dosage. The results of the experiment showed that the optimum removal percentage and adsorbing capacity can be reached when about 1 g l^−1^ dosage was added into the metal solutions. So the optimum quantity of adsorbent dosage was selected as 1 g l^−1^ for further experiments.

#### 3.2.2. Effect of contact time and initial metal concentration on adsorption kinetics

In the present study, the applicability of the pseudo-first-order and pseudo-second-order model was tested for the adsorption of toxic metals onto four kinds of biochars. Moreover, the pseudo-first-order kinetic model predicted a significantly lower value of the equilibrium adsorption capacity (*q_t_*) than the experimental value, which is not shown here but indicates the inapplicability of this model. The adsorption data were then analysed using the pseudo-second-order kinetic model, which provided a much better fit with the data than the first-order model and strongly fitted with the experimental data.

According to the pseudo-second-order kinetic model (electronic supplementary material, figures S4–S7), the initial metal concentration had a pronounced effect on its removal from aqueous solutions. It was found that the calculated amount of Pb(II) adsorption increased from 9.49 to 26.87 mg g^−1^, 9.40 to 28.18 mg g^−1^, 9.62 to 28.06 mg g^−1^ and 9.53 to 28.70 mg g^−1^ with increasing initial concentration of Pb(II) from 10 ppm to 30 ppm for LEC200, LEC300, LEC400 and LEC500, respectively. Correspondingly, the amount of Zn(II) increased from 8.83 to 12.27 mg g^−1^, 9.86 to 21.87 mg g^−1^, 9.74 to 30.70 mg g^−1^ and 9.84 to 29.48 mg g^−1^. The amount of Cu(II) was from 5.54 to 19.62 mg g^−1^, 7.76 to 24.21 mg g^−1^, 9.39 to 30.10 mg g^−1^ and 9.95 to 30.12 mg g^−1^, and that of Cd(II) was from 5.40 to 18.26 mg g^−1^, 9.03 to 27.06 mg g^−1^, 11.10 to 30.20 mg g^−1^ and 9.66 to 29.37 mg g^−1^. It was also found that adsorption increased with increasing contact time at all initial metal concentrations and equilibrium was attained within 30 min. All kinetic parameters including the linear correlation coefficient (R^2^) obtained from the fitting model plots with experimental data under different conditions were very high and the theoretical *q_t_* was closer to the experimental *q_t_* (in the electronic supplementary material). The values of the rate constant *k*_2_ almost decreased with initial metal concentration. The reason for this behaviour may be due to the lower competition for the sorption sites at a lower concentration. At higher concentrations, the competition for the surface active sites will be high and consequently lower sorption rates were obtained. Similar types of kinetic model parameters were obtained by various researchers for a few other observations systems reported in the literature [43–46]. This was because the initial metal concentration provides the driving force to overcome the resistance to the mass transfer of toxic metals between the aqueous and the solid phase. Under the same conditions, if the concentration of heavy metal in solution increased, then the active sites on the biomass would be surrounded by many more metal ions, so that more sorption would occur [47]. In view of all these results, the amounts of metal adsorption increased with the enhancement of calcinations temperatures and improved significantly for LEC400 and LEC500. What's more, the equilibrium was attained more quickly with the increase in calcinations temperatures.

#### 3.2.3. Adsorption isotherms and thermodynamic studies

**Figure 6.**
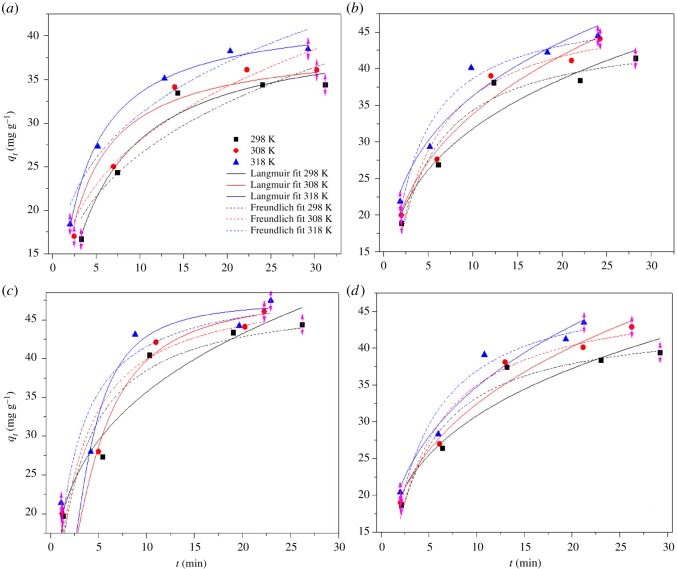
Adsorption isotherms of four metal ions onto LEC500 at 298, 308 and 318 K. (*a*) Pb(II), (*b*) Zn(II), (*c*) Cu(II), (*d*) Cd(II).

LEC500 of 1 g l^−1^ was added to 20, 40, 60, 80 and 100 mg l^−1^ metal solutions to research the adsorption isotherms at different temperatures of 298, 308 and 318 K. The results in figure 6 indicated that the Langmuir model can represent Pb(II), Zn(II), Cu(II) and Cd(II) sorption processes more reasonably than the Freundlich model. The calculated *q*_max_ values (39.09 mg g^−1^ for Pb(II), 45.40 mg g^−1^ for Zn(II), 48.20 mg g^−1^ for Cu(II) and 44.04 mg g^−1^ for Cd(II)) by the Langmuir model were higher than the experimental data (34.41 mg g^−1^ for Pb(II), 41.23 mg g^−1^ for Zn(II), 44.17 mg g^−1^ for Cu(II) and 39.81 mg g^−1^ for Cd(II)) at 298 K. The fractional value of 1/*n* (0 < 1/*n* < 1) obtained for the metal sorption system based on the Freundlich model clearly indicated the active sites of LEC500 were heterogeneous for metal binding.

Another noteworthy observation was that the sorption capacities of four metals by LEC500 were slightly increased with the elevation of temperature, which implied the endothermic nature of the sorption process on the biochar. This may be due to an increase in the mobility of the dye molecules and an increase in the number of active sites for the adsorption with increasing temperature [40]. The Gibb's free energy (Δ*G*^0^), entropy (ΔS^0^) and enthalpy (Δ*H*^0^) changed for metal adsorption had been determined by the application of equations (2.5), (2.6) and (2.7). The positive values of Δ*H*^0^ confirmed the endothermic nature of adsorption. The endothermic adsorption on other adsorbent systems has also been reported [48,49]. The positive value of Δ*S*^0^ suggests increased randomness at the solid solution interface occurs in the internal structure of the adsorption of metals onto LEC500. Negative Δ*G*^0^ values validated that the sorption process of four toxic metals onto LEC500 were all essentially spontaneous. The decrease in Δ*G*^0^ with increasing temperature showed the adsorption of metal was favourable at a higher temperature.

As shown in table 1, the sorption capacities of four toxic metal ions onto LEC500 were higher than the other listed lignocellulosic materials which indicated LEC500 had higher exploitability and utilization prospects for dealing with metal wastewater.

**Table 1.** Reported sorption capacities for several lignocellulosic materials.

adsorbent	Pb (mg g^−1^)	Zn (mg g^−1^)	Cu (mg g^−1^)	Cd (mg g^−1^)	refs
LEC500	39.09	45.40	48.20	44.04	this article
wheat straw	3.11	—	4.48	9.96	[50]
soya bean stem	6.83	—	5.44	2.02	[50]
corn straw	3.93	—	2.18	10.75	[50]
oat straw	18.84	—	5.18	4.70	[50]
tea biochar	33.49	—	16.87	11.83	[51]
*Hizikia* biochar	10.39	10.56	—	14.42	[52]
hardwood biochar	—	4.54	6.79	—	[53]
corn straw biochar	—	11.0	12.52	—	[53]
peanut shell biochar	22.82	—	—	—	[18]
*Miscanthus* biochar	—	—	—	13.24	[54]

## 4. Conclusion

The biochars produced from long-root *Eichhornia crassipes* at 200, 300, 400 and 500°C (LEC200, LEC300, LEC400 and LEC500) were able to remove Pb(II), Zn(II), Cu(II) and Cd(II) efficiently, but the LEC500 was the best one, which can be seen from the results of SEM, BET and elemental analyser. It was also found that the alkyl, carboxyl, phosphate and cyano groups in biochars can play a role in adsorbing metals. Furthermore, the adsorption data provided a much better fit to the pseudo-second-order kinetic model which strongly fitted with the experimental data and the Langmuir model can represent Pb(II), Zn(II), Cu(II) and Cd(II) sorption processes more reasonably than the Freundlich model. The positive and negative values of Δ*H*^0^ and Δ*G*^0^ confirm the endothermic and spontaneous nature of adsorption.

## Supplementary Material

ESM 1 - BET for LEC200

## Supplementary Material

ESM 2 - BET for LEC300

## Supplementary Material

ESM 3 - BET for LEC400

## Supplementary Material

ESM 4 - BET for LEC500

## Supplementary Material

ESM 5 - Adsorption isotherms and thermodynamic studies

## Supplementary Material

ESM 6 - Effect of adsorbent dosage on adsorption

## Supplementary Material

ESM 7 - Effect of contact time and initial metal concentration on adsorption kinetics

## Supplementary Material

ESM 8 - FT-IR spectroscopy materials for LEC200

## Supplementary Material

ESM 9 - FT-IR spectroscopy materials for LEC300

## Supplementary Material

ESM 10 - FT-IR spectroscopy materials for LEC400

## Supplementary Material

ESM 11 - FT-IR spectroscopy materials for LEC500

## Data accessibility

Our datas are deposited at the electronic supplementary material

## References

[1] Wang Z, Hou L, Liu Y, Wang Y, Ma LQ 2018 Metal contamination in a riparian wetland: distribution, fractionation and plant uptake. Chemosphere 200, 587–593. (10.1016/j.chemosphere.2018.02.159) 29505931

[2] Tabasi S, Hassani H, Azadmehr AR 2018 Phytoextraction-based process of metal absorption from soil in mining areas (tailing dams) by *Medicago sativa L*. (Alfalfa) (Case study: Sarcheshmeh porphyry copper mine, SE of Iran). J. Min. Environ. Saf. 157, 102–110. (doi:10.22044/jme.2017.897)

[3] Hwang SK, Jho EH 2018 Heavy metal and sulfate removal from sulfate-rich synthetic mine drainages using sulfate reducing bacteria. Sci. Total Environ. 635, 1308–1316. (10.1016/j.scitotenv.2018.04.231) 29710584

[4] Hu X, Li Y, Wang Y, Li X, Li H, Liu X 2010 Adsorption kinetics, thermodynamics and isotherm of thiacalix[4]arene-loaded resin to heavy metal ions. Desalination 259, 76–83. (10.1016/j.desal.2010.04.032)

[5] Torma CZ, Csefalvay E 2018 Nanofiltration and electrodialysis: alternatives in heavy metal containing high salinity process water treatment. Chem. Pap. 72, 1115–1124. (10.1007/s11696-018-0433-7)

[6] Li L, Dong J, Nenoff T 2007 Transport of water and alkali metal ions through MFI zeolite membranes during reverse osmosis.*Sep**.* Purif. Technol. 53, 42–48. (10.1016/j.seppur.2006.06.012)

[7] Ipec U 2005 Removal of Ni(II) and Zn(II) from an aqueous solution by reverse osmosis. Desalination 174, 161–169. (10.1016/j.desal.2004.09.009)

[8] Ellwood BB, Benoist SL, Hassani AE, Wheeler C, Crick RE 2004 Impact ejecta layer from the mid-Devonian: possible connection to global mass extinctions. Science 303, 1734–1737. (10.1126/science.1091164) 12805544

[9] Dabrowski A, Hubicki Z, Podkoscielny P, Robens E 2004 Selective removal of the heavy metal ions from waters and industrial wastewaters by ion-exchange method. Chemosphere 56, 91–106. (10.1016/j.chemosphere.2004.03.006) 15120554

[10] Altun T, Pehlivan E 2012 Removal of Cr(VI) from aqueous solutions by modified walnut shells. Food Chem. 132, 693–700. (10.1016/j.foodchem.2011.10.099)

[11] Serencam H, Gundogdu A, Uygur Y, Kemer B, Bulut VN, Duran C, Soylak M, Tufekci M 2008 Removal of cadmium from aqueous solution by Nordmann fir (*Abies nordmanniana* (Stev.) Spach. Subsp. nordmanniana) leaves. Bioresour. Technol. 99, 1992–2000. (10.1016/j.biortech.2007.03.021) 17475482

[12] Gupta N, Amritphale SS, Chandra N 2010 Removal of Zn (II) from aqueous solution by using hybrid precursor of silicon and carbon. Bioresour. Technol. 101, 3355–3362. (10.1016/j.biortech.2009.12.024) 20100656

[13] Xu X, Cao X, Zhao L, Wang H, Yu H, Gao B 2013 Removal of Cu, Zn, and Cd from aqueous solutions by the dairy manure-derived biochar. Environ. Sci. Pollut. 20, 358–368. (10.1007/s11356-012-0873-5) 22477163

[14] Mansour MS, Ossman ME, Farag HA 2011 Removal of Cd (II) ion from waste water by adsorption onto polyaniline coated on sawdust. Desalination 272, 301–305. (10.1016/j.desal.2011.01.037)

[15] Šćiban M, Klašnja M, Škrbić B 2008 Adsorption of copper ions from water by modified agricultural by-products. Desalination 229, 170–180. (10.1016/j.desal.2007.08.017)

[16] Jiang J, Xu RK, Jiang TY, Li Z 2012 Immobilization of Cu(II), Pb(II) and Cd(II) by the addition of rice straw derived biochar to a simulated polluted Ultisol. J. Hazard. Mater. 229–230, 145–150. (10.1016/j.jhazmat.2012.05.086) 22704774

[17] Inyang M, Gao B, Yao Y, Xue Y, Zimmerman AR 2012 Removal of heavy metals from aqueous solution by biochars derived from anaerobically digested biomass. Bioresour. Technol. 110, 50–56. (10.1016/j.biortech.2012.01.072) 22325901

[18] Chen X, Chen G, Chen L, Chen Y, Lehmann J, McBride MB, Hay AG 2011 Adsorption of copper and zinc by biochars produced from pyrolysis of hardwood and corn straw in aqueous solution. Bioresour. Technol. 102, 8877–8884. (10.1016/j.biortech.2011.06.078) 21764299

[19] Cao XD, Ma LN, Gao B, Environ Willie H. 2009 Dairy-manure derived biochar effectively sorbs lead and atrazine. Environ. Sci.Technol. 43, 3285–3291. (10.1021/es803092k) 19534148

[20] Lu H, Zhang W, Yang Y, Huang X, Wang S, Qiu R 2012 Relative distribution of Pb^2+^ sorption mechanisms by sludge-derived biochar. Water. Res. 46, 854–862. (10.1016/j.watres.2011.11.058) 22189294

[21] Cha JS, Park SH, Jung SC, Ryu C, Jeon JK, Shin MC, Park YK 2016 Production and utilization of biochar: a review. J. Ind. Eng. Chem. 40, 1–15. (10.1016/j.jiec.2016.06.002)

[22] Lee HW, Kim YM, Kim S, Ryu C, Park SH, Park YK 2018 Review of the use of activated biochar for energy and environmental applications. Carbon Lett. 26, 1–10. (10.5714/CL.2018.26.001)

[23] Lee H et al. 2016 Adsorptive removal of atmospheric pollutants over *Pyropia tenera* chars. Carbon Lett. 19, 79–88. (10.5714/CL.2016.19.07)

[24] Lee HW, Park RS, Park SH, Jung SC, Jeon JK, Kim SC, Chung JD, Choi WG, Park YK 2016 Cu^2+^ ion reduction in wastewater over RDF-derived char. Carbon Lett. 18, 49–55. (10.5714/CL.2016.18.049)

[25] Wang H, Xia W, Lu P 2017 Study on adsorption characteristics of biochars on heavy metals in soil. Korean J. Chem. Eng. 34, 1867–1873. (10.1007/s11814-017-0048-7)

[26] Ahmadi M, Kouhgardi E, Ramavandi B 2016 Physico-chemical study of dew melon peel biochar for chromium attenuation from simulated and actual wastewaters. Korean J. Chem. Eng. 33, 2589–2601. (10.1007/s11814-016-0135-1)

[27] Gwenzi W, Chaukura N, Noubactep C, Mukome FND 2017 Biochar-based water treatment as a potential low-cost and sustainable technology for clean water provision. J. Environ. Manage. 197, 732–749. (10.1016/j.jenvman.2017.03.087) 28454068

[28] Lund BC, Abrams TE, Gravely AA 2011 Validity of PTSD diagnoses in VA administrative data: comparison of VA administrative PTSD diagnoses to self-reported PTSD Checklist scores. J. Rehabil. Res. Dev. 48, 21–30. (10.1682/JRRD.2009.08.0116) 21328160

[29] Cardwell AJ, Hawker DW, Greenway M 2002 Metal accumulation in aquatic macrophytes from southeast Queensland, Australia. Chemosphere 48, 653–663. (10.1016/S0045-6535(02)00164-9) 12201195

[30] Miretzky P, Saralegui A, Cirelli AF 2004 Aquatic macrophytes potential for the simultaneous removal of heavy metals (Buenos Aires, Argentina). Chemosphere 57, 997–1005. (10.1016/j.chemosphere.2004.07.024) 15488590

[31] Oki S, Ma Y 1983 Removal of some heavy metals from polluted water by water hyacinth (*Eichhornia crassipes*). Bull. Environm. Conta. Toxicol 30, 170–177. (10.1007/BF01610117) 6839042

[32] Ismail Z, Beddri AM 2008 Potential of water hyacinth as a removal agent for heavy metals from petroleum refinery effluents. Water, Air, Soil Pollut. 199, 57–65. (10.1007/s11270-008-9859-9)

[33] Agunbiade FO, Olu-Owolabi BI, Adebowale KO 2009 Phytoremediation potential of *Eichornia crassipes* in metal-contaminated coastal water. Bioresour. Technol. 100, 4521–4526. (10.1016/j.biortech.2009.04.011) 19414252

[34] Mahamadi C, Nharingo T 2010 Competitive adsorption of Pb^2+^, Cd^2+^ and Zn^2+^ ions onto *Eichhornia crassipes* in binary and ternary systems. Bioresour. Technol. 101, 859–864. (10.1016/j.biortech.2009.08.097) 19773154

[35] Mishra VK, Tripathi BD 2009 Accumulation of chromium and zinc from aqueous solutions using water hyacinth (*Eichhornia crassipes*). J. Hazard. Mater. 164, 1059–1063. (10.1016/j.jhazmat.2008.09.020) 18938031

[36] Mahamadi C, Zambara P 2013 High Cu removal from water using water hyacinth fixed on alginate. Environ. Chem. Lett. 11, 377–383. (10.1007/s10311-013-0418-2)

[37] Miretzky P, Saralegui A, Fernández Cirelli A 2006 Simultaneous heavy metal removal mechanism by dead macrophytes. Chemosphere 62, 247–254. (10.1016/j.chemosphere.2005.05.010) 15990152

[38] Gupta VK, Rastogi A 2008 Biosorption of lead from aqueous solutions by green algae *Spirogyra* species: kinetics and equilibrium studies. J. Hazard. Mater. 152, 407–414. (10.1016/j.jhazmat.2007.07.028) 17716814

[39] John Peter AL, Viraraghavan T 2008 Removal of thallium from aqueous solutions by modified *Aspergillus niger* biomass. Bioresour. Technol. 99, 618–625. (10.1016/j.biortech.2006.12.038) 17376677

[40] Bhainsa KC, D'Souza SF 2008 Removal of copper ions by the filamentous fungus, *Rhizopus oryzae* from aqueous solution. Bioresour. Technol. 99, 3829–3835. (10.1016/j.biortech.2007.07.032) 17804218

[41] Arias F, Sen TK 2009 Removal of zinc metal ion (Zn^2+^) from its aqueous solution by kaolin clay mineral: a kinetic and equilibrium study. Colloids Surf. A 348, 100–108. (10.1016/j.colsurfa.2009.06.036)

[42] Zhang K, Cheung WH, Valix M 2005 Roles of physical and chemical properties of activated carbon in the adsorption of lead ions. Chemosphere 60, 1129–1140. (10.1016/j.chemosphere.2004.12.059) 15993162

[43] Ofomaja AE, Naidoo EB 2011 Biosorption of copper from aqueous solution by chemically activated pine cone: a kinetic study. Chem. Eng. J. 175, 260–270. (10.1016/j.cej.2011.09.103)

[44] Ofomaja AE 2010 Intraparticle diffusion process for lead(II) biosorption onto mansonia wood sawdust. Bioresour. Technol. 101, 5868–5876. (10.1016/j.biortech.2010.03.033) 20385492

[45] Gupta SS, Bhattacharyya KG 2011 Kinetics of adsorption of metal ions on inorganic materials: a review. Adv. Colloid Interfac. 162, 39–58. (10.1016/j.cis201012.004) 21272842

[46] Sen TK, Afroze S, Ang HM 2010 Equilibrium, kinetics and mechanism of removal of methylene blue from aqueous solution by adsorption onto pine cone biomass of *Pinus radiata*. Water, Air, Soil Pollut. 218, 499–515. (10.1007/s11270-010-0663-y)

[47] Saeed A, Iqba Ml, Akhtar MW 2005 Removal and recovery of lead(II) from single and multimetal (Cd, Cu, Ni, Zn) solutions by crop milling waste (black gram husk). J. Hazard Mater. 117, 65–73. (10.1016/j.jhazmat.2004.09.008) 15621354

[48] Namasivayam C, Kavitha D 2002 Removal of Congo Red from water by adsorption onto activated carbon prepared from coir pith, an agricultural solid waste. Dyes Pigm. 54, 47–58. (10.1016/S0143-7208(02)00025-6)

[49] Benyoucef S, Amrani M 2011 Adsorption of phosphate ions onto low cost Aleppo pine adsorbent. Desalination 275, 231–236. (10.1016/j.desal.2011.03.004)

[50] Kołodyńska D, Wnętrzak R, Leahy J, Hayes M, Kwapiński W, Hubicki Z 2012 Kinetic and adsorptive characterization of biochar in metal ions removal. Chem. Eng. J. 197, 295–305. (10.1016/j.cej.2012.05.025)

[51] Wan S, Ma Z, Xue Y, Ma M, Xu S, Qian L, Zhang Q 2014 Sorption of lead (II), cadmium (II), and copper (II) ions from aqueous solutions using tea waste. Ind. Eng. Chem. Res. 53, 3629–3635. (10.1016/j.cej.2012.05.025)

[52] Shin WS, Kim YK 2014 Biosorption characteristics of heavy metals (Ni^2+^, Zn^2+^, Cd^2+^, Pb^2+^) from aqueous solution by *Hizikia fusiformis*. Environ. Earth Sci. 71, 4107–4114. (10.1007/s12665-013-2799-8)

[53] Xue Y, Gao B, Yao Y, Inyang M, Zhang M, Zimmerman AR, Ro KS 2012 Hydrogen peroxide modification enhances the ability of biochar (hydrochar) produced from hydrothermal carbonization of peanut hull to remove aqueous heavy metals: batch and column tests. Chem. Eng. J. 200, 673–680. (10.1016/j.cej.2012.06.116)

[54] Kim WK, Shim T, Kim YS, Hyun S, Ryu C, Park YK, Jung J 2013 Characterization of cadmium removal from aqueous solution by biochar produced from a giant *Miscanthus* at different pyrolytic temperatures. Bioresour. Technol. 138, 266–270. (10.1016/j.biortech.2013.03.186) 23619139

