# Supplementary material for: Removal of toxic metals from aqueous solution by biochars derived from long-root Eichhornia crassipes
Source: R Soc Open Sci. 2018 Oct 24;5(10):180966. doi: 10.1098/rsos.180966 (PMC6227962; doi:10.1098/rsos.180966)
Supplement: ESM 1 - BET for LEC200 [file rsos180966supp1.docx]

Quantachrome NovaWin - Data Acquisition and Reduction

for NOVA instruments

?1994-2010, Quantachrome Instruments

version 11.0

Analysis Report

Operator:open Date:2014/10/06 Operator:open Date:10/7/2014

Sample ID: L1 Filename: C:\QCdata\Physisorb\sttn_A_20141005-L1.qps

Sample Desc: Comment:

Sample weight: 0.0893 g Sample Volume: 0.06071 cc

Outgas Time: 0.0 hrs OutgasTemp: 0.0 C

Analysis gas: Nitrogen Bath Temp: 77.3 K

Press. Tolerance:0.100/0.100 (ads/des)Equil time: 60/60 sec (ads/des) Equil timeout: 240/240 sec (ads/des)

Analysis Time: 402.7 min End of run: 2014/10/06 2:12:27 Instrument: Nova Station A

Cell ID: 1 F/W version: 0.00

Adsorbate Nitrogen Temperature 77.350K

Molec. Wt.: 28.013 g Cross Section: 16.200 Ų Liquid Density: 0.808 g/cc

Surface Area Data

MultiPoint BET 1.498e+00 m?g

Langmuir surface area 2.276e+00 m?g

BJH method cumulative adsorption surface area 6.867e+00 m?g

BJH method cumulative desorption surface area 9.598e+00 m?g

DH method cumulative adsorption surface area 8.081e+00 m?g

DH method cumulative desorption surface area 9.738e+00 m?g

t-method external surface area 1.498e+00 m?g

Pore Volume Data

Total pore volume for pores with Diameter

less than 184.88 nm at P/Po = 0.989516 9.162e-03 cc/g

BJH method cumulative adsorption pore volume 1.092e-02 cc/g

BJH method cumulative desorption pore volume 1.119e-02 cc/g

DH method cumulative adsorption pore volume 1.095e-02 cc/g

DH method cumulative desorption pore volume 1.096e-02 cc/g

HK method cumulative pore volume 7.080e-04 cc/g

SF method cumulative pore volume 7.522e-04 cc/g

Pore Size Data

Average pore Diameter 2.447e+01 nm

BJH method adsorption pore Diameter (Mode Dv(d)) 2.683e+00 nm

BJH method desorption pore Diameter (Mode Dv(d)) 2.172e+00 nm

DH method adsorption pore Diameter (Mode Dv(d)) 2.683e+00 nm

DH method desorption pore Diameter (Mode Dv(d)) 2.172e+00 nm

HK method pore Diameter (Mode) 9.825e-01 nm

SF method pore Diameter (Mode) 1.772e+00 nm

Quantachrome NovaWin - Data Acquisition and Reduction

for NOVA instruments

?1994-2010, Quantachrome Instruments

version 11.0

Analysis Report

Operator:open Date:2014/10/06 Operator:open Date:10/7/2014

Sample ID: L1 Filename: C:\QCdata\Physisorb\sttn_A_20141005-L1.qps

Sample Desc: Comment:

Sample weight: 0.0893 g Sample Volume: 0.06071 cc

Outgas Time: 0.0 hrs OutgasTemp: 0.0 C

Analysis gas: Nitrogen Bath Temp: 77.3 K

Press. Tolerance:0.100/0.100 (ads/des)Equil time: 60/60 sec (ads/des) Equil timeout: 240/240 sec (ads/des)

Analysis Time: 402.7 min End of run: 2014/10/06 2:12:27 Instrument: Nova Station A

Cell ID: 1 F/W version: 0.00

Adsorbate Nitrogen Temperature 77.350K

Molec. Wt.: 28.013 g Cross Section: 16.200 Ų Liquid Density: 0.808 g/cc

Average Pore Size summary

Average pore Diameter = 2.44717e+01 nm

Quantachrome NovaWin - Data Acquisition and Reduction

for NOVA instruments

?1994-2010, Quantachrome Instruments

version 11.0

Analysis Report

Operator:open Date:2014/10/06 Operator:open Date:10/7/2014

Sample ID: L1 Filename: C:\QCdata\Physisorb\sttn_A_20141005-L1.qps

Sample Desc: Comment:

Sample weight: 0.0893 g Sample Volume: 0.06071 cc

Outgas Time: 0.0 hrs OutgasTemp: 0.0 C

Analysis gas: Nitrogen Bath Temp: 77.3 K

Press. Tolerance:0.100/0.100 (ads/des)Equil time: 60/60 sec (ads/des) Equil timeout: 240/240 sec (ads/des)

Analysis Time: 402.7 min End of run: 2014/10/06 2:12:27 Instrument: Nova Station A

Cell ID: 1 F/W version: 0.00

Adsorbate Nitrogen Temperature 77.350K

Molec. Wt.: 28.013 g Cross Section: 16.200 Ų Liquid Density: 0.808 g/cc

Relative Volume @ STP

Pressure

cc/g

7.73600e-03 0.0861

1.38760e-02 0.1509

2.42010e-02 0.2232

3.41130e-02 0.2751

4.43010e-02 0.2928

5.42060e-02 0.3078

1.07090e-01 0.3820

1.56538e-01 0.4635

2.07429e-01 0.6001

2.30698e-01 0.6854

2.54311e-01 0.7660

2.79871e-01 0.8584

3.05439e-01 0.9620

3.27250e-01 1.0607

3.53177e-01 1.1702

3.80432e-01 1.2857

4.05778e-01 1.4044

4.56670e-01 1.6266

5.04888e-01 1.8593

5.56490e-01 1.9566

6.05464e-01 2.0754

6.55324e-01 2.2731

7.07750e-01 2.5231

7.58245e-01 2.8300

8.08976e-01 3.3369

8.58607e-01 3.8204

9.10445e-01 4.3134

9.55662e-01 4.8757

9.89516e-01 5.9231

9.48759e-01 5.0123

9.04022e-01 4.5788

8.52276e-01 4.1603

8.03615e-01 3.7565

7.52525e-01 3.3123

7.02894e-01 3.0811

6.49780e-01 2.8565

6.04464e-01 2.6965

5.73825e-01 2.6412

5.47616e-01 2.6167

5.25996e-01 2.5870

5.01077e-01 2.5478

4.76823e-01 2.4328

4.51920e-01 2.3206

4.23022e-01 2.1897

4.00326e-01 1.3901

3.51642e-01 1.1742

3.02659e-01 0.9601

2.52188e-01 0.7659

1.91575e-01 0.5685

1.51368e-01 0.4670

9.96750e-02 0.3787

6.87610e-02 0.3341

5.49390e-02 0.3156

2.78280e-02 0.2461

Quantachrome NovaWin - Data Acquisition and Reduction

for NOVA instruments

?1994-2010, Quantachrome Instruments

version 11.0

Analysis Report

Operator:open Date:2014/10/06 Operator:open Date:10/7/2014

Sample ID: L1 Filename: C:\QCdata\Physisorb\sttn_A_20141005-L1.qps

Sample Desc: Comment:

Sample weight: 0.0893 g Sample Volume: 0.06071 cc

Outgas Time: 0.0 hrs OutgasTemp: 0.0 C

Analysis gas: Nitrogen Bath Temp: 77.3 K

Press. Tolerance:0.100/0.100 (ads/des)Equil time: 60/60 sec (ads/des) Equil timeout: 240/240 sec (ads/des)

Analysis Time: 402.7 min End of run: 2014/10/06 2:12:27 Instrument: Nova Station A

Cell ID: 1 F/W version: 0.00

Adsorbate Nitrogen Temperature 77.350K

Molec. Wt.: 28.013 g Cross Section: 16.200 Ų Liquid Density: 0.808 g/cc

Total Pore Volume summary

Total Pore Volume

Total pore volume = 9.162e-03 cc/g

for pores smaller than 184.9 nm (Diameter)

at P/Po = 0.98952
