# Supplementary material for: Removal of toxic metals from aqueous solution by biochars derived from long-root Eichhornia crassipes
Source: R Soc Open Sci. 2018 Oct 24;5(10):180966. doi: 10.1098/rsos.180966 (PMC6227962; doi:10.1098/rsos.180966)
Supplement: ESM 2 - BET for LEC300 [file rsos180966supp2.docx]

Quantachrome NovaWin - Data Acquisition and Reduction

for NOVA instruments

?1994-2010, Quantachrome Instruments

version 11.0

Analysis Report

Operator:open Date:2014/10/06 Operator:open Date:10/7/2014

Sample ID: L2 Filename: C:\QCdata\Physisorb\sttn_B_20141005-L2.qps

Sample Desc: Comment:

Sample weight: 0.1233 g Sample Volume: 0.28289 cc

Outgas Time: 0.0 hrs OutgasTemp: 0.0 C

Analysis gas: Nitrogen Bath Temp: 77.3 K

Press. Tolerance:0.100/0.100 (ads/des)Equil time: 60/60 sec (ads/des) Equil timeout: 240/240 sec (ads/des)

Analysis Time: 504.5 min End of run: 2014/10/06 2:14:18 Instrument: Nova Station B

Cell ID: 2 F/W version: 0.00

Adsorbate Nitrogen Temperature 77.350K

Molec. Wt.: 28.013 g Cross Section: 16.200 Ų Liquid Density: 0.808 g/cc

Surface Area Data

MultiPoint BET 5.594e+01 m?g

Langmuir surface area 3.661e+01 m?g

BJH method cumulative adsorption surface area 7.226e+01 m?g

BJH method cumulative desorption surface area 7.525e+01 m?g

DH method cumulative adsorption surface area 7.353e+01 m?g

DH method cumulative desorption surface area 7.675e+01 m?g

t-method external surface area 5.594e+01 m?g

Pore Volume Data

Total pore volume for pores with Diameter

less than 104.69 nm at P/Po = 0.981291 5.516e-02 cc/g

BJH method cumulative adsorption pore volume 7.294e-02 cc/g

BJH method cumulative desorption pore volume 7.350e-02 cc/g

DH method cumulative adsorption pore volume 7.156e-02 cc/g

DH method cumulative desorption pore volume 7.220e-02 cc/g

HK method cumulative pore volume 8.264e-03 cc/g

SF method cumulative pore volume 9.015e-03 cc/g

Pore Size Data

Average pore Diameter 3.944e+00 nm

BJH method adsorption pore Diameter (Mode Dv(d)) 2.259e+00 nm

BJH method desorption pore Diameter (Mode Dv(d)) 2.165e+00 nm

DH method adsorption pore Diameter (Mode Dv(d)) 2.259e+00 nm

DH method desorption pore Diameter (Mode Dv(d)) 2.165e+00 nm

HK method pore Diameter (Mode) 1.827e+00 nm

SF method pore Diameter (Mode) 3.350e+00 nm

Quantachrome NovaWin - Data Acquisition and Reduction

for NOVA instruments

?1994-2010, Quantachrome Instruments

version 11.0

Analysis Report

Operator:open Date:2014/10/06 Operator:open Date:10/7/2014

Sample ID: L2 Filename: C:\QCdata\Physisorb\sttn_B_20141005-L2.qps

Sample Desc: Comment:

Sample weight: 0.1233 g Sample Volume: 0.28289 cc

Outgas Time: 0.0 hrs OutgasTemp: 0.0 C

Analysis gas: Nitrogen Bath Temp: 77.3 K

Press. Tolerance:0.100/0.100 (ads/des)Equil time: 60/60 sec (ads/des) Equil timeout: 240/240 sec (ads/des)

Analysis Time: 504.5 min End of run: 2014/10/06 2:14:18 Instrument: Nova Station B

Cell ID: 2 F/W version: 0.00

Adsorbate Nitrogen Temperature 77.350K

Molec. Wt.: 28.013 g Cross Section: 16.200 Ų Liquid Density: 0.808 g/cc

Average Pore Size summary

Average pore Diameter = 3.94404e+00 nm

Quantachrome NovaWin - Data Acquisition and Reduction

for NOVA instruments

?1994-2010, Quantachrome Instruments

version 11.0

Analysis Report

Operator:open Date:2014/10/06 Operator:open Date:10/7/2014

Sample ID: L2 Filename: C:\QCdata\Physisorb\sttn_B_20141005-L2.qps

Sample Desc: Comment:

Sample weight: 0.1233 g Sample Volume: 0.28289 cc

Outgas Time: 0.0 hrs OutgasTemp: 0.0 C

Analysis gas: Nitrogen Bath Temp: 77.3 K

Press. Tolerance:0.100/0.100 (ads/des)Equil time: 60/60 sec (ads/des) Equil timeout: 240/240 sec (ads/des)

Analysis Time: 504.5 min End of run: 2014/10/06 2:14:18 Instrument: Nova Station B

Cell ID: 2 F/W version: 0.00

Adsorbate Nitrogen Temperature 77.350K

Molec. Wt.: 28.013 g Cross Section: 16.200 Ų Liquid Density: 0.808 g/cc

Relative Volume @ STP

Pressure

cc/g

6.40700e-03 0.3186

1.24560e-02 0.5575

2.19020e-02 0.9057

3.19830e-02 1.2662

4.18260e-02 1.6159

5.32820e-02 2.0079

9.91100e-02 3.5620

1.45747e-01 5.1211

1.95325e-01 6.7722

2.28491e-01 7.8876

2.50233e-01 8.6216

2.77153e-01 9.5334

3.02805e-01 10.4083

3.22874e-01 11.0896

3.50817e-01 12.0484

3.77408e-01 12.9604

4.00983e-01 13.7702

4.48659e-01 15.4184

4.95541e-01 17.0494

5.52076e-01 19.0267

5.96697e-01 20.6013

6.52018e-01 22.5764

6.99686e-01 24.2936

7.45354e-01 25.9554

8.04699e-01 28.1430

8.48908e-01 29.8111

8.98051e-01 31.7316

9.51706e-01 34.0001

9.81291e-01 35.6590

9.48499e-01 34.0658

9.02319e-01 32.1891

8.53857e-01 30.3352

8.02851e-01 28.4618

7.38359e-01 26.1348

7.02000e-01 24.8459

6.48634e-01 22.9444

6.00599e-01 21.2429

5.72733e-01 20.2805

5.53832e-01 19.6317

5.27103e-01 18.7082

4.99840e-01 17.7532

4.74769e-01 16.8849

4.50621e-01 16.0543

4.21304e-01 15.0402

4.00104e-01 13.7535

3.36505e-01 11.5648

2.98879e-01 10.2629

2.50378e-01 8.6459

1.90667e-01 6.6608

1.50131e-01 5.3122

1.04257e-01 3.7568

6.86850e-02 2.5243

5.43660e-02 2.0833

2.72560e-02 1.1302 Quantachrome NovaWin - Data Acquisition and Reduction

for NOVA instruments

?1994-2010, Quantachrome Instruments

version 11.0

Analysis Report

Operator:open Date:2014/10/06 Operator:open Date:10/7/2014

Sample ID: L2 Filename: C:\QCdata\Physisorb\sttn_B_20141005-L2.qps

Sample Desc: Comment:

Sample weight: 0.1233 g Sample Volume: 0.28289 cc

Outgas Time: 0.0 hrs OutgasTemp: 0.0 C

Analysis gas: Nitrogen Bath Temp: 77.3 K

Press. Tolerance:0.100/0.100 (ads/des)Equil time: 60/60 sec (ads/des) Equil timeout: 240/240 sec (ads/des)

Analysis Time: 504.5 min End of run: 2014/10/06 2:14:18 Instrument: Nova Station B

Cell ID: 2 F/W version: 0.00

Adsorbate Nitrogen Temperature 77.350K

Molec. Wt.: 28.013 g Cross Section: 16.200 Ų Liquid Density: 0.808 g/cc

Total Pore Volume summary

Total Pore Volume

Total pore volume = 5.516e-02 cc/g

for pores smaller than 104.7 nm (Diameter)

at P/Po = 0.98129
