# Supplementary material for: Removal of toxic metals from aqueous solution by biochars derived from long-root Eichhornia crassipes
Source: R Soc Open Sci. 2018 Oct 24;5(10):180966. doi: 10.1098/rsos.180966 (PMC6227962; doi:10.1098/rsos.180966)
Supplement: ESM 3 - BET for LEC400 [file rsos180966supp3.docx]

Quantachrome NovaWin - Data Acquisition and Reduction

for NOVA instruments

?1994-2010, Quantachrome Instruments

version 11.0

Analysis Report

Operator:open Date:2014/10/07 Operator:open Date:10/7/2014

Sample ID: L3 Filename: C:\QCdata\Physisorb\sttn_B_20141006-L3.qps

Sample Desc: Comment:

Sample weight: 0.0514 g Sample Volume: 0.02151 cc

Outgas Time: 0.0 hrs OutgasTemp: 0.0 C

Analysis gas: Nitrogen Bath Temp: 77.3 K

Press. Tolerance:0.100/0.100 (ads/des)Equil time: 60/60 sec (ads/des) Equil timeout: 240/240 sec (ads/des)

Analysis Time: 405.7 min End of run: 2014/10/07 15:29:47 Instrument: Nova Station B

Cell ID: 4 F/W version: 0.00

Adsorbate Nitrogen Temperature 77.350K

Molec. Wt.: 28.013 g Cross Section: 16.200 Ų Liquid Density: 0.808 g/cc

Surface Area Data

MultiPoint BET 2.212e+00 m?g

Langmuir surface area 2.092e+00 m?g

BJH method cumulative adsorption surface area 8.453e+00 m?g

BJH method cumulative desorption surface area 1.037e+01 m?g

DH method cumulative adsorption surface area 1.005e+01 m?g

DH method cumulative desorption surface area 1.053e+01 m?g

t-method external surface area 2.212e+00 m?g

Pore Volume Data

Total pore volume for pores with Diameter

less than 116.89 nm at P/Po = 0.983284 1.329e-02 cc/g

BJH method cumulative adsorption pore volume 1.589e-02 cc/g

BJH method cumulative desorption pore volume 1.542e-02 cc/g

DH method cumulative adsorption pore volume 1.591e-02 cc/g

DH method cumulative desorption pore volume 1.509e-02 cc/g

HK method cumulative pore volume 5.575e-04 cc/g

SF method cumulative pore volume 2.745e-05 cc/g

Pore Size Data

Average pore Diameter 2.404e+01 nm

BJH method adsorption pore Diameter (Mode Dv(d)) 3.185e+00 nm

BJH method desorption pore Diameter (Mode Dv(d)) 2.432e+00 nm

DH method adsorption pore Diameter (Mode Dv(d)) 3.185e+00 nm

DH method desorption pore Diameter (Mode Dv(d)) 2.432e+00 nm

HK method pore Diameter (Mode) 1.337e+00 nm

SF method pore Diameter (Mode) 3.239e+00 nm

Quantachrome NovaWin - Data Acquisition and Reduction

for NOVA instruments

?1994-2010, Quantachrome Instruments

version 11.0

Analysis Report

Operator:open Date:2014/10/07 Operator:open Date:10/7/2014

Sample ID: L3 Filename: C:\QCdata\Physisorb\sttn_B_20141006-L3.qps

Sample Desc: Comment:

Sample weight: 0.0514 g Sample Volume: 0.02151 cc

Outgas Time: 0.0 hrs OutgasTemp: 0.0 C

Analysis gas: Nitrogen Bath Temp: 77.3 K

Press. Tolerance:0.100/0.100 (ads/des)Equil time: 60/60 sec (ads/des) Equil timeout: 240/240 sec (ads/des)

Analysis Time: 405.7 min End of run: 2014/10/07 15:29:47 Instrument: Nova Station B

Cell ID: 4 F/W version: 0.00

Adsorbate Nitrogen Temperature 77.350K

Molec. Wt.: 28.013 g Cross Section: 16.200 Ų Liquid Density: 0.808 g/cc

Average Pore Size summary

Average pore Diameter = 2.40380e+01 nm

Quantachrome NovaWin - Data Acquisition and Reduction

for NOVA instruments

?1994-2010, Quantachrome Instruments

version 11.0

Analysis Report

Operator:open Date:2014/10/07 Operator:open Date:10/7/2014

Sample ID: L3 Filename: C:\QCdata\Physisorb\sttn_B_20141006-L3.qps

Sample Desc: Comment:

Sample weight: 0.0514 g Sample Volume: 0.02151 cc

Outgas Time: 0.0 hrs OutgasTemp: 0.0 C

Analysis gas: Nitrogen Bath Temp: 77.3 K

Press. Tolerance:0.100/0.100 (ads/des)Equil time: 60/60 sec (ads/des) Equil timeout: 240/240 sec (ads/des)

Analysis Time: 405.7 min End of run: 2014/10/07 15:29:47 Instrument: Nova Station B

Cell ID: 4 F/W version: 0.00

Adsorbate Nitrogen Temperature 77.350K

Molec. Wt.: 28.013 g Cross Section: 16.200 Ų Liquid Density: 0.808 g/cc

Relative Volume @ STP

Pressure

cc/g

7.99600e-03 0.1347

1.43440e-02 0.1964

2.47290e-02 0.2698

3.44360e-02 0.3164

4.50430e-02 0.3267

5.46500e-02 0.3340

1.04108e-01 0.3473

1.57733e-01 0.3665

2.07306e-01 0.4878

2.30502e-01 0.5605

2.55285e-01 0.6703

2.82833e-01 0.7763

3.09088e-01 0.9007

3.30812e-01 0.9904

3.55872e-01 1.1233

3.82743e-01 1.2554

4.06832e-01 1.3961

4.57815e-01 1.7037

5.06780e-01 2.0268

5.56992e-01 2.3516

6.04694e-01 2.6981

6.57618e-01 3.1128

7.08755e-01 3.5529

7.53283e-01 3.9904

7.99659e-01 4.4522

8.56235e-01 5.0731

9.04491e-01 5.7259

9.54834e-01 6.8721

9.83284e-01 8.5949

9.48148e-01 7.1996

8.99945e-01 6.2646

8.47221e-01 5.6329

7.97926e-01 5.1514

7.50779e-01 4.7439

6.99881e-01 4.3307

6.45308e-01 3.9247

6.00064e-01 3.5811

5.79780e-01 3.4757

5.54227e-01 3.3144

5.24529e-01 3.1342

4.98121e-01 2.9730

4.74894e-01 2.8280

4.45844e-01 2.6328

4.25860e-01 2.5235

3.93358e-01 1.3092

3.47806e-01 1.1015

2.96623e-01 0.8124

2.46418e-01 0.6407

2.02176e-01 0.4806

1.48273e-01 0.3733

9.59960e-02 0.3551

6.67450e-02 0.3404

5.36250e-02 0.3304

3.49750e-02 0.3223 Quantachrome NovaWin - Data Acquisition and Reduction

for NOVA instruments

?1994-2010, Quantachrome Instruments

version 11.0

Analysis Report

Operator:open Date:2014/10/07 Operator:open Date:10/7/2014

Sample ID: L3 Filename: C:\QCdata\Physisorb\sttn_B_20141006-L3.qps

Sample Desc: Comment:

Sample weight: 0.0514 g Sample Volume: 0.02151 cc

Outgas Time: 0.0 hrs OutgasTemp: 0.0 C

Analysis gas: Nitrogen Bath Temp: 77.3 K

Press. Tolerance:0.100/0.100 (ads/des)Equil time: 60/60 sec (ads/des) Equil timeout: 240/240 sec (ads/des)

Analysis Time: 405.7 min End of run: 2014/10/07 15:29:47 Instrument: Nova Station B

Cell ID: 4 F/W version: 0.00

Adsorbate Nitrogen Temperature 77.350K

Molec. Wt.: 28.013 g Cross Section: 16.200 Ų Liquid Density: 0.808 g/cc

Total Pore Volume summary

Total Pore Volume

Total pore volume = 1.329e-02 cc/g

for pores smaller than 116.9 nm (Diameter)

at P/Po = 0.98328
