# Supplementary material for: Removal of toxic metals from aqueous solution by biochars derived from long-root Eichhornia crassipes
Source: R Soc Open Sci. 2018 Oct 24;5(10):180966. doi: 10.1098/rsos.180966 (PMC6227962; doi:10.1098/rsos.180966)
Supplement: ESM 4 - BET for LEC500 [file rsos180966supp4.docx]

Quantachrome NovaWin - Data Acquisition and Reduction

for NOVA instruments

?1994-2010, Quantachrome Instruments

version 11.0

Analysis Report

Operator:open Date:2014/10/07 Operator:open Date:10/7/2014

Sample ID: L4 Filename: C:\QCdata\Physisorb\sttn_B_20141005-L4.qps

Sample Desc: Comment:

Sample weight: 0.0757 g Sample Volume: 0 cc

Outgas Time: 0.0 hrs OutgasTemp: 0.0 C

Analysis gas: Nitrogen Bath Temp: 77.3 K

Press. Tolerance:0.100/0.100 (ads/des)Equil time: 60/60 sec (ads/des) Equil timeout: 240/240 sec (ads/des)

Analysis Time: 450.3 min End of run: 2014/10/07 21:39:17 Instrument: Nova Station B

Cell ID: 4 F/W version: 0.00

Adsorbate Nitrogen Temperature 77.350K

Molec. Wt.: 28.013 g Cross Section: 16.200 Ų Liquid Density: 0.808 g/cc

Surface Area Data

MultiPoint BET 3.008e+00 m?g

Langmuir surface area 1.373e+00 m?g

BJH method cumulative adsorption surface area 8.736e+00 m?g

BJH method cumulative desorption surface area 1.011e+01 m?g

DH method cumulative adsorption surface area 8.857e+00 m?g

DH method cumulative desorption surface area 1.034e+01 m?g

t-method external surface area 3.008e+00 m?g

Pore Volume Data

Total pore volume for pores with Diameter

less than 166.71 nm at P/Po = 0.988355 1.629e-02 cc/g

BJH method cumulative adsorption pore volume 1.914e-02 cc/g

BJH method cumulative desorption pore volume 1.723e-02 cc/g

DH method cumulative adsorption pore volume 1.869e-02 cc/g

DH method cumulative desorption pore volume 1.689e-02 cc/g

HK method cumulative pore volume 1.737e-04 cc/g

SF method cumulative pore volume 3.921e-01 cc/g

Pore Size Data

Average pore Diameter 2.166e+01 nm

BJH method adsorption pore Diameter (Mode Dv(d)) 3.202e+00 nm

BJH method desorption pore Diameter (Mode Dv(d)) 2.427e+00 nm

DH method adsorption pore Diameter (Mode Dv(d)) 3.202e+00 nm

DH method desorption pore Diameter (Mode Dv(d)) 2.427e+00 nm

HK method pore Diameter (Mode) 1.407e+00 nm

SF method pore Diameter (Mode) 3.820e+00 nm

Quantachrome NovaWin - Data Acquisition and Reduction

for NOVA instruments

?1994-2010, Quantachrome Instruments

version 11.0

Analysis Report

Operator:open Date:2014/10/07 Operator:open Date:10/7/2014

Sample ID: L4 Filename: C:\QCdata\Physisorb\sttn_B_20141005-L4.qps

Sample Desc: Comment:

Sample weight: 0.0757 g Sample Volume: 0 cc

Outgas Time: 0.0 hrs OutgasTemp: 0.0 C

Analysis gas: Nitrogen Bath Temp: 77.3 K

Press. Tolerance:0.100/0.100 (ads/des)Equil time: 60/60 sec (ads/des) Equil timeout: 240/240 sec (ads/des)

Analysis Time: 450.3 min End of run: 2014/10/07 21:39:17 Instrument: Nova Station B

Cell ID: 4 F/W version: 0.00

Adsorbate Nitrogen Temperature 77.350K

Molec. Wt.: 28.013 g Cross Section: 16.200 Ų Liquid Density: 0.808 g/cc

Average Pore Size summary

Average pore Diameter = 2.16557e+01 nm

Quantachrome NovaWin - Data Acquisition and Reduction

for NOVA instruments

?1994-2010, Quantachrome Instruments

version 11.0

Analysis Report

Operator:open Date:2014/10/07 Operator:open Date:10/7/2014

Sample ID: L4 Filename: C:\QCdata\Physisorb\sttn_B_20141005-L4.qps

Sample Desc: Comment:

Sample weight: 0.0757 g Sample Volume: 0 cc

Outgas Time: 0.0 hrs OutgasTemp: 0.0 C

Analysis gas: Nitrogen Bath Temp: 77.3 K

Press. Tolerance:0.100/0.100 (ads/des)Equil time: 60/60 sec (ads/des) Equil timeout: 240/240 sec (ads/des)

Analysis Time: 450.3 min End of run: 2014/10/07 21:39:17 Instrument: Nova Station B

Cell ID: 4 F/W version: 0.00

Adsorbate Nitrogen Temperature 77.350K

Molec. Wt.: 28.013 g Cross Section: 16.200 Ų Liquid Density: 0.808 g/cc

Relative Volume @ STP

Pressure

cc/g

8.06600e-03 0.0820

1.44960e-02 0.1165

2.48670e-02 0.1541

3.46990e-02 0.1918

4.55160e-02 0.2011

5.50340e-02 0.2054

1.05773e-01 0.2074

1.54492e-01 0.2706

2.06407e-01 0.3894

2.27287e-01 0.4618

2.54949e-01 0.5618

2.83119e-01 0.6859

3.09445e-01 0.8118

3.30379e-01 0.9220

3.57919e-01 1.0626

3.83991e-01 1.2291

4.10317e-01 1.4145

4.60657e-01 1.6994

5.07917e-01 1.9886

5.52987e-01 2.2967

6.06453e-01 2.6662

6.57500e-01 3.0684

7.06811e-01 3.4532

7.58057e-01 3.9284

8.03535e-01 4.4246

8.58676e-01 5.0318

9.08601e-01 5.8104

9.56659e-01 7.1803

9.88355e-01 10.5290

9.51528e-01 8.2526

9.01718e-01 7.1711

8.47483e-01 6.4859

8.01530e-01 6.0399

7.46788e-01 5.5023

6.95111e-01 5.0882

6.47890e-01 4.6988

5.97358e-01 4.2929

5.78407e-01 4.2038

5.43799e-01 3.9677

5.26738e-01 3.8806

5.03615e-01 3.7535

4.75030e-01 3.5669

4.54719e-01 3.4747

4.24544e-01 3.3009

3.98195e-01 1.3558

3.45551e-01 1.0225

2.96056e-01 0.7502

2.45063e-01 0.5532

1.96006e-01 0.3606

1.59003e-01 0.2803

9.99960e-02 0.2001

6.67450e-02 0.2044

5.36250e-02 0.2021

3.39750e-02 0.1885 Quantachrome NovaWin - Data Acquisition and Reduction

for NOVA instruments

?1994-2010, Quantachrome Instruments

version 11.0

Analysis Report

Operator:open Date:2014/10/07 Operator:open Date:10/7/2014

Sample ID: L4 Filename: C:\QCdata\Physisorb\sttn_B_20141005-L4.qps

Sample Desc: Comment:

Sample weight: 0.0757 g Sample Volume: 0 cc

Outgas Time: 0.0 hrs OutgasTemp: 0.0 C

Analysis gas: Nitrogen Bath Temp: 77.3 K

Press. Tolerance:0.100/0.100 (ads/des)Equil time: 60/60 sec (ads/des) Equil timeout: 240/240 sec (ads/des)

Analysis Time: 450.3 min End of run: 2014/10/07 21:39:17 Instrument: Nova Station B

Cell ID: 4 F/W version: 0.00

Adsorbate Nitrogen Temperature 77.350K

Molec. Wt.: 28.013 g Cross Section: 16.200 Ų Liquid Density: 0.808 g/cc

Total Pore Volume summary

Total Pore Volume

Total pore volume = 1.629e-02 cc/g

for pores smaller than 166.7 nm (Diameter)

at P/Po = 0.98835
